# AraPheno and the AraGWAS Catalog 2020: a major database update including RNA-Seq and knockout mutation data for *Arabidopsis thaliana*

**DOI:** 10.1093/nar/gkz925

**Published:** 2019-10-23

**Authors:** Matteo Togninalli, Ümit Seren, Jan A Freudenthal, J Grey Monroe, Dazhe Meng, Magnus Nordborg, Detlef Weigel, Karsten Borgwardt, Arthur Korte, Dominik G Grimm

**Affiliations:** 1 Machine Learning and Computational Biology Lab, Department of Biosystems Science and Engineering, ETH Zürich, Basel, Switzerland; 2 Swiss Institute of Bioinformatics, Basel, Switzerland; 3 Gregor Mendel Institute of Molecular Plant Biology, Vienna, Austria; 4 Center for Computational and Theoretical Biology, University Würzburg, Würzburg, Germany; 5 Max Planck Institute for Developmental Biology, Tübingen, Germany; 6 Google, Mountain View, USA; 7 Technical University of Munich, TUM Campus Straubing for Biotechnology and Sustainability, Bioinformatics, Straubing, Germany; 8 Weihenstephan-Triesdorf University of Applied Sciences, Bioinformatics, Straubing, Germany

## Abstract

Genome-wide association studies (GWAS) are integral for studying genotype-phenotype relationships and gaining a deeper understanding of the genetic architecture underlying trait variation. A plethora of genetic associations between distinct loci and various traits have been successfully discovered and published for the model plant *Arabidopsis thaliana*. This success and the free availability of full genomes and phenotypic data for more than 1,000 different natural inbred lines led to the development of several data repositories. AraPheno (https://arapheno.1001genomes.org) serves as a central repository of population-scale phenotypes in *A. thaliana*, while the AraGWAS Catalog (https://aragwas.1001genomes.org) provides a publicly available, manually curated and standardized collection of marker-trait associations for all available phenotypes from AraPheno. In this major update, we introduce the next generation of both platforms, including new data, features and tools. We included novel results on associations between knockout-mutations and all AraPheno traits. Furthermore, AraPheno has been extended to display RNA-Seq data for hundreds of accessions, providing expression information for over 28 000 genes for these accessions. All data, including the imputed genotype matrix used for GWAS, are easily downloadable via the respective databases.

## INTRODUCTION

*Arabidopsis thaliana* is a naturally inbred plant, which means that all lines have nearly completely homozygous genomes. This property enables the study of diverse phenotypes under different and, more importantly, controlled environmental conditions of genetically identical plants (1). *Arabidopsis thaliana* is a prime model system in plant biology and beyond (2). The free availability of high-quality full genome genotype data for more than a thousand individuals provides a unique platform for reproducible research (3). These resources have enabled the development and benchmarking of novel tools in different communities, e.g. in machine learning and data mining (4–6).

Genome-wide association studies (GWAS) became an essential tool for unfolding genotype-phenotype relationships. For this purpose, a number of tools and statistical methods have been developed (7). Many GWAS have been conducted successfully for hundreds of *A. thaliana* phenotypes within the last view years (1,8–17). Till recently, phenotypic data and GWAS results were fragmented across different websites. To structure this vast amount of phenotypic data, we developed and published AraPheno, a central database to collect and organise population-scale phenotypes for *A. thaliana* (18). Similarly, we developed the AraGWAS Catalog, a central and manually curated resource of genetic associations for all available phenotypes from AraPheno (19), similar to the NHGRI-EBI GWAS Catalog (20). More importantly, all GWAS results in the AraGWAS Catalog are re-computed using a permutation-based and standardized GWAS pipeline on an identical release of genomic data to enable comparative analysis between GWAS results and phenotypes as well as to detect pleiotropic effects. When combined with the availability of genotypes, we have a resource analogous to the UK Biobank (21). Despite the comparatively small number of phenotypes and sample sizes in AraPheno, the benefits are that those are all from the same cohort and could easily be combined for meta-analyses.

We extensively updated both platforms, AraPheno (https://arapheno.1001genomes.org) and the AraGWAS Catalog (https://aragwas.1001genomes.org). We revised both database backends and added new data and features—many that have been requested by the community. Furthermore, we developed a completely new branch to provide RNA-Seq expression profiles for thousands of samples and genes in AraPheno, as well as novel associations between knockout (KO) mutations and population-scale phenotypes in the AraGWAS Catalog.

## UPDATES IN ARAPHENO

We extended AraPheno and developed a completely new branch to not only store phenotypic data, but to also provide gene expression data from RNA-Seq experiments (22,23) for hundreds of accessions and thousands of genes (Table 1, https://arapheno.1001genomes.org/rnaseq). At the moment we only provide the expression data in AraPheno, but in principle, gene expression data could be treated as high-dimensional phenotypic data and thus could be used directly for GWAS as well as for transcription-wide association studies (TWAS) (24), where the transcript abundances are treated as explanatory variables. We added a switch to the landing page, where users can easily change between the two branches, phenotype-related and RNA-Seq-related data. Different color codings of the web interface indicate on which of the branches the user currently is (brown for phenotypes and blue for RNA-Seq). Similar to the initial phenotypic views, the AraPheno RNA-Seq views also contain a study overview table, as well as a detailed gene view summarizing gene specific meta-information and expression values across all available study samples.

**Table 1.** AraPheno and AraGWAS Catalog data content and summary statistics as of 6 September 2019

**Data Content**		
**AraPheno**	**Data Statistics 2017**	**Data Statistics 2019**
Studies	6	22
Phenotypes	260	462
Phenotype Values	52 741	193 616
Phenotyped Accessions	1425	1496
RNA-Seq Studies	-	2
Accessions with RNA Seq data	-	788
Unique RNA-Seq Genes	-	28 819
Total RNA-Seq Expression Values	-	20 371 657

**AraGWAS**	**Data Statistics 2018**	**Data Statistics 2019**
SNP-Trait Associations at *P* < 10^−4^	222 983	1 152 968
Significant SNP Associations (Bonferroni)	9527	104 874
Significant SNP Associations (Permutation)	3887	44 680
KO-Mutations	-	2088
KO-Trait Associations at *P* < 10^−4^	-	319
Significant KO Associations (Bonferroni)	-	130
Significant KO Associations (Permutation)	-	15

For many statistical analyses, phenotypic or gene expression values need to be normalized if they do not match the assumptions of the statistical test employed or if expensive permutation-based experiments cannot be performed. For this purpose, we added a new tool to directly transform any phenotype by applying different transformations (e.g. log, anscombe, box-cox).

In the last few years the number of studies in AraPheno has markedly increased (Table 1). New population-scale phenotypes were submitted either actively by the community or by the authors (15–17,25–31). As of September 2019, AraPheno contains 462 published phenotypes for *A. thaliana* (Table 1), making it the largest data resource for publicly available population-scale phenotypes in *A. thaliana*, by far. We made it easier to cite the publicly available studies providing the original phenotypes by clicking on the new ‘Cite’ link at the respective individual study or phenotype view (see online FAQ, https://arapheno.1001genomes.org/faq/cite/). This link includes references to the original study, the respective phenotype/study DOI and *AraPheno*.

A new Master Accession Table (https://arapheno.1001genomes.org/accessions/) summarizes information from all *A. thaliana* accessions where seeds are available. This table provides meta-information (e.g. geographic positions) and information about public genotype releases (e.g. RegMap, 1001 Genomes, Fully Imputed of 2029 accession) for the respective accession. In addition, we provide a counter which indicates the number of phenotypes the accession is part of. The table can be filtered by these different genotype releases and all data are easily downloadable.

Finally, we performed major changes on the backend to enable increased performance for multiple users, including the download and upload of large amounts of phenotypes. Further, we now provide the possibility to download the full AraPheno database as a single ZIP file, including all phenotypes and its meta-information. The online FAQ gives a detailed overview of the content of the ZIP file (https://arapheno.1001genomes.org/faq/content/).

## UPDATES IN THE ARAGWAS CATALOG

All GWAS results in the AraGWAS Catalog have been systematically re-computed on all publicly available population-scale phenotypes from AraPheno using GWAS-Flow, a fast implementation based on linear mixed models for efficient genome-wide association studies that easily enables the use of permutation-based GWAS thresholds (32). For the genotype data the same SNP-Matrix of 2029 accessions on 10 709 466 segregating markers has been used, as reported in (19). Permutation-based significance thresholds are computed and reported for every phenotype to account for non-Gaussian phenotypic distributions. This standardized GWAS pipeline and the identical genotype release will enable maximum comparability between different phenotypes and GWAS experiments and might thus enable the detection of pleiotropic effects. Table 1 gives an overview about the latest update compared to the initial publication in 2018 (19). Furthermore, we now also provide effect-size estimated and standard-errors for all associations.

**Figure 1.**
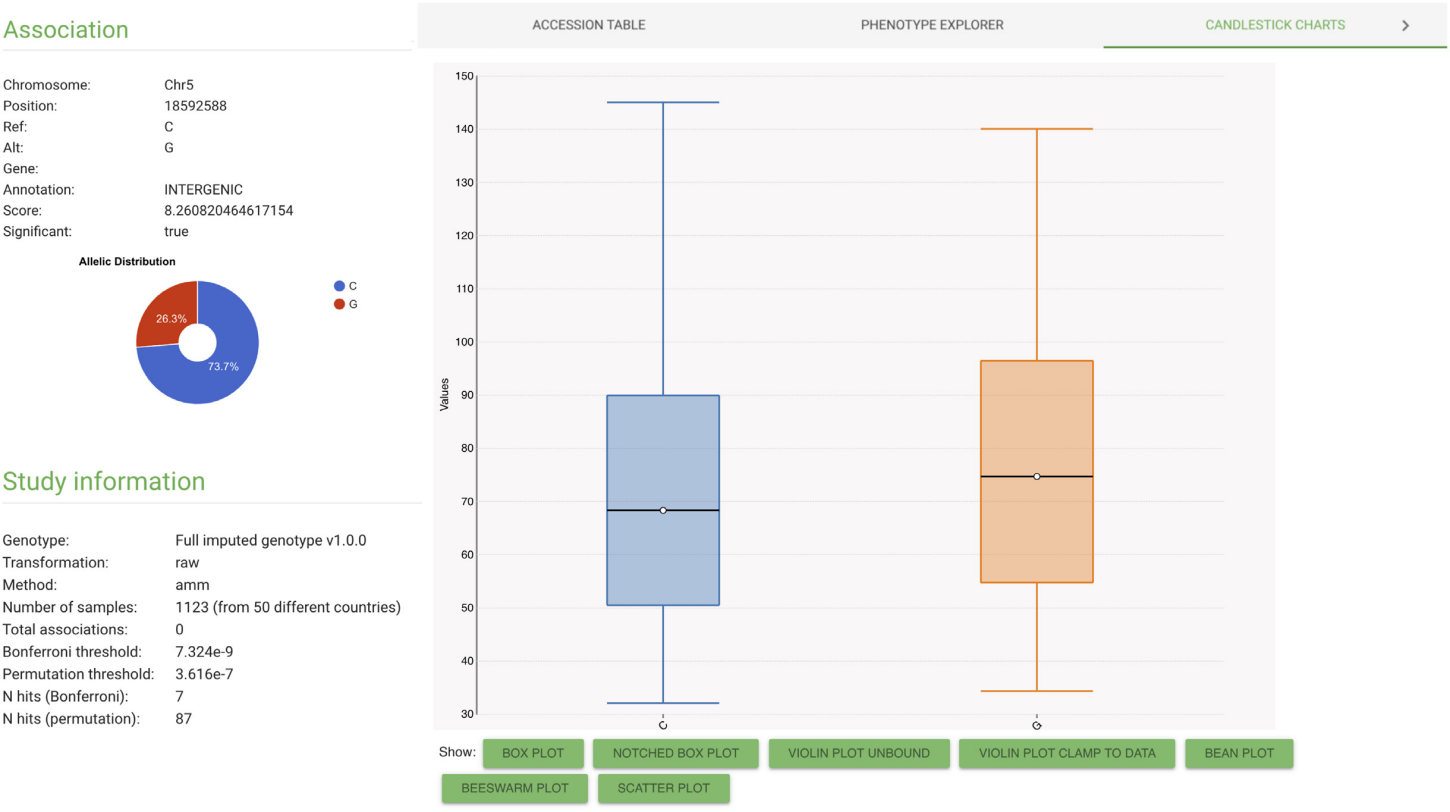
*Detailed association view*. This view shows detailed information about the alleles and the allelic distribution of phenotypic values for a selected association. The association table gives individual information about each sample, the allele and phenotype value as well as all associated meta-information (e.g. geographic coordinates). All plots can be adjusted dynamically and data can be exported in CSV format directly within the browser (https://aragwas.1001genomes.org/#/study/262/associations/5_18592588).

For downstream analyses it is not only of interest to consider *P*-values, but more importantly also to look at effect sizes and its standard errors, as well as allelic information and phenotypic distributions of the different allelic groups. Therefore, we now include the calculation of the effect sizes into our re-analysis and implemented a detailed individual association view (Figure 1). This view can be easily accessed for every single association in the AraGWAS Catalog by clicking on the chromosome position identifier (e.g. Chr4:1269036, https://aragwas.1001genomes.org/#/study/144/associations/4_1269036) of any association. The view provides necessary summary statistics and dynamic visualizations illustrating the phenotypic distributions of the different allelic groups (Figure 1). Furthermore, various filters can be used to adjust the visualizations, e.g. to filter by geographic origin. An accession table in the detailed association view, shows the phenotypic value for each accession as well as its allele.

Loss-of-function mutations can be an important source of genetic variation in the evolution of plant traits (33,34). Thus, we extended the GWAS analysis to compute associations between reported knockout (KO) mutations based on loss-of-function alleles of full genes (35) and all publicly available phenotypes in *A. thaliana*. For this purpose, we created additional views in the AraGWAS Catalog to report these results. The ‘Top KO Mutation’ view provides a full list of all significant associations between KO genes and phenotypes (https://aragwas.1001genomes.org/#/top-ko-mutations). The ‘Top KO Genes’ view shows all top associated KO mutation genes, and indicates if any of the KO genes is associated to more than one phenotype (https://aragwas.1001genomes.org/#/top-ko-genes). Furthermore, we updated each detailed study view to not only show interactive Manhattan plots for SNP-trait associations, but to also show interactive Manhattan plots for KO-Trait associations (https://doi.org/10.21958/gwas:50). All dots in the Manhattan plots are interactive and show additional information about the association, when moving the mouse over the association. When clicking at one of the association dots, the user will be redirected to a detailed gene view (https://aragwas.1001genomes.org/#/gene/AT1G57570). In the next section, we provide a more detailed analysis of these KO association results.

Finally, a Download Center (https://aragwas.1001genomes.org/#/download-center) has been added which allows the download of the full database, including all associations, as well as the download of imputed genotype data or KO mutation data for maximum reproducibility of all results or followup analysis.

## NOVEL KNOCKOUT-MUTATION ASSOCIATIONS WITH PHENOTYPES

**Figure 2.**
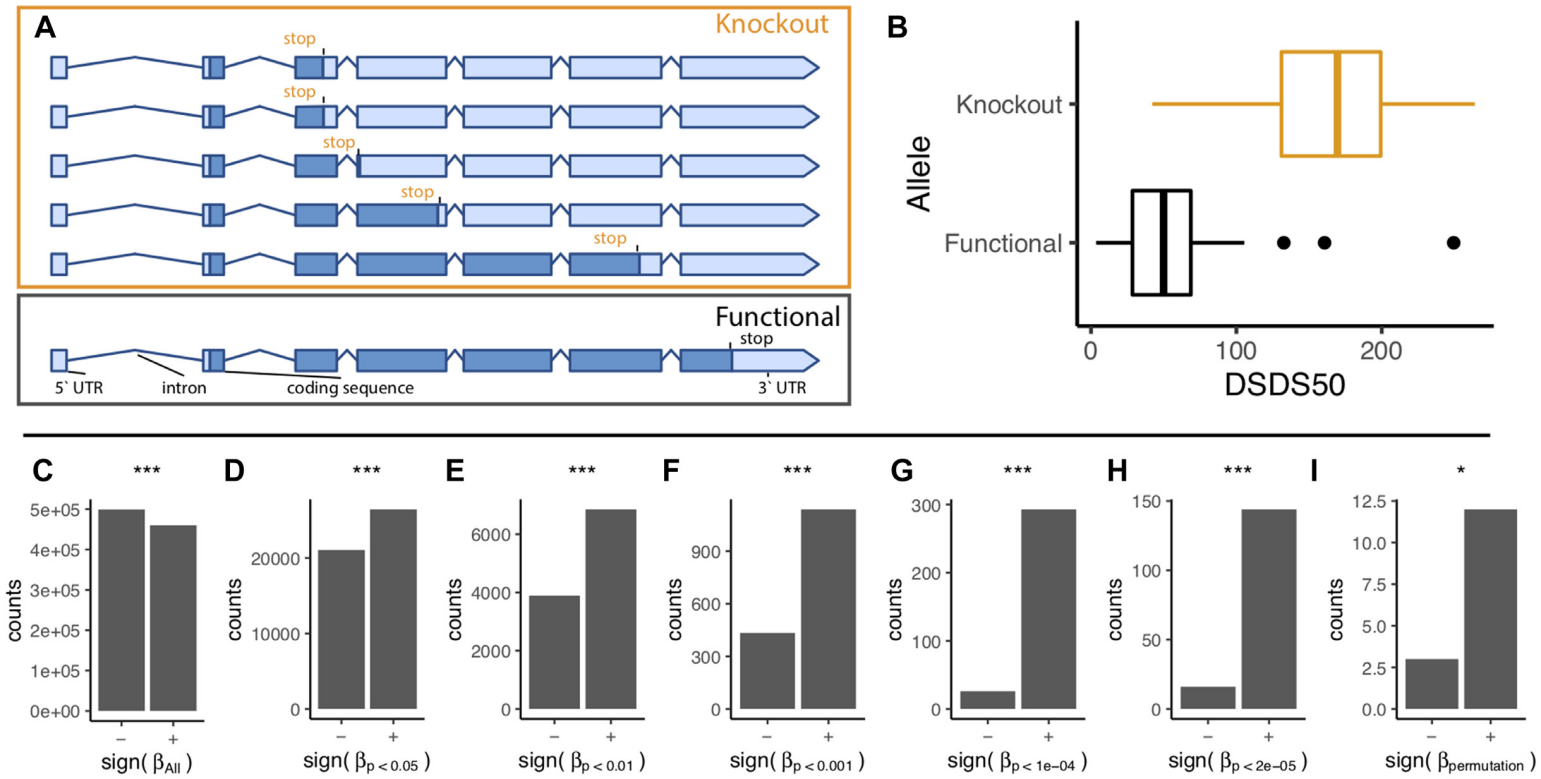
KO-trait association detected between AT1G57570 and days of seed dry storage required to reach 50% germination (DSDS50) reported by (1). (**A**) Predicted natural knockout alleles identified in (35). (**B**) Boxplots showing DSDS50 for accessions with functional (black) versus KO alleles (orange) of AT1G57570. The vertical lines mark the medians while the boxes indicate the interquartile ranges (IQR) between the 25th and 75th quantiles, and the whiskers mark no >1.5 IQR. More broadly, there was a tendency for significant KO allele associations to have positive beta coefficients. Bar plots show the relative frequency of positive and negative beta (β) coefficients across all traits tested (***P < 2 × 10^−16^, *P < 0.05, Chi-squared tests), for (**C**) all associations regardless of significance and for associations subsetted by varying significance thresholds: (**D**) *P* < 0.05, (**E**) *P* < 0.01, (**F**) *P* < 0.001, (**G**) *P* < 1 × 10^−4^, (**H**) *P* < 2.4 × 10^−5^ (threshold after Bonferroni correction) (**I**) permutation-based significance thresholds.

We analyzed associations between traits and natural KO (knockout) mutations based on loss-of-function alleles reported by (35). These analyses revealed associations undetected by SNP based GWAS. For example, we found that, after permutation based threshold filtering, natural KO alleles in AT1G57570 (https://aragwas.1001genomes.org/#/gene/AT1G57570), a mannose-binding lectin superfamily protein that is highly expressed during seed germination, were associated (*P* < 2.3 × 10^−7^, β = 99.7) with ‘number of days of seed dry storage required to reach 50% germination’ (DSDS50, https://doi.org/10.21958/phenotype:50) as reported by (1) (Figure 2). Interestingly, when looking at broad patterns of KO-trait associations, regardless of the significance of associations, we found an overrepresentation of KO-trait relationships toward smaller trait values (greater number of negative β coefficients, Figure 2C). In contrast, when subsetting associations by those meeting various significance thresholds, we observed a significant enrichment in knockout alleles associated with increased trait values (greater number of positive β coefficients, Figure 2D–I). This observation may reflect the fact that reference gene models are based on the Col-0 genotype, which is relatively small and rapid cycling. Such results are consistent with previously observed biases in the direction of knockout effects (35,36) and motivate efforts to define gene functional allele state informed by diverse genotypes.

## DATA & CODE ACCESSIBILITY

All data, including phenotypes, summary statistics and meta-information can be downloaded directly within the browser using the respective buttons. Both databases come with fully integrated Representational State Transfer (REST) web service interfaces. These REST interfaces allow users to easily interact with both databases programmatically and to download the data in various common formats. Further, the REST interfaces allow the user to filter the data before download. Detailed API descriptions and examples on how to use the REST endpoints can be found in the online FAQ of AraPheno (https://arapheno.1001genomes.org/faq/rest/) and the AraGWAS Catalog (https://aragwas.1001genomes.org/docs/). Furthermore we provide the ability to download the full databases (including all available information, meta-data und summary statistics).

To enable easy trackability and citability of phenotypic data, as well as of individual studies and GWAS results, all data entries are linked to the respective original publications and are associated with individual and persistent DOIs (provided by DataCite, https://datacite.org).

Both platforms are implemented in Python using modern web technologies. The backend is based on the Django web-framework (https://www.djangoproject.com) and the web interface is implemented using HTML5 and Javascript. The web interface of AraGWAS is built using Vue.js (https://vuejs.org/), a single-page app development framework. The web interface uses REST to retrieve data from the backend which is built with Django (https://www.djangoproject.com/) and Django REST framework (https://www.django-rest-framework.org/). AraPheno uses a server side rendered approach based on Django. The interactive parts in the web interface are implemented using jQuery (https://jquery.com/). The code for both platforms is open-source and freely available on GitHub for download, as well as to submit feedback or issues (AraPheno: https://github.com/1001genomes/AraPheno; AraGWAS Catalog: https://github.com/1001genomes/AraGWAS).

## CONCLUSIONS AND FUTURE DIRECTIONS

AraPheno and the AraGWAS Catalog are the most comprehensive and manually curated databases of publicly available population-scale phenotypes and standardized GWAS results for the model system *A. thaliana*. The databases have evolved to be central resources for the growing *A. thaliana* community (31,37–39). Researchers from the *A. thaliana* community use AraPheno either to publish newly measured phenotypes (15–17,27–31) or use the publicly available data to generate new biological insights (40), as demonstrated in AraCLIM. In addition both platforms are of great value for method development and benchmarking in other areas, such as bioinformatics, and machine learning (4–6).

With this introduction of the next generation of both resources, we extensively revised the database backends, web interfaces, added novel features (e.g. RNA-Seq views, KO-Associations, detailed association views) and new data (Table 1). We showed that associations between KO-mutations (35) and phenotypes are an additional and highly valuable type of information for the plant biology community.

Recently, Watanabe *et al.* (41) released an overview of pleiotropic and genetic effects of complex traits in humans. Due to the standardized pipeline we used, the *A. thaliana* data simplify the study of pleiotropic effects across various experiments and phenotypes. Additionally, fast GWAS implementations enable the recomputation of associations for all phenotypes whenever a new genotype matrix will be released. We plan to continuously update both resources, but we also invite the scientific community to upload their phenotypic data to AraPheno simultaneously with publishing their manuscripts. All seeds for the different accessions are readily available via the *Arabidopsis* stock centers ABRC (https://abrc.osu.edu/) and NASC (http://arabidopsis.info/), which enables everyone to generate new data and contribute to our resources. Additionally, we also simplified the data submission and provide an extensive tutorial. In future releases, we plan to integrate more diverse types of phenotypes and meta-information, similar to what we did for the RNA-Seq data. One example would be the recently published climatic data (40) for all *A. thaliana* accessions that allow to link environmental variables with different genotypes and/or phenotypes. Currently, we only added a link to the AraCLIM portal but plan an easy and smooth integration of such data in future releases.

Our vision would be that anyone generating population-scale phenotypes in *A. thaliana* will upload their phenotypic data to AraPheno. This will not only make AraPheno and the AraGWAS Catalog more valuable resources but will also set a precedent for the deposition of these data. Additionally, it will also help these studies to gain additional visibility. In the long term this will facilitate investigations into more complex research questions as any researcher can easily access all data in one location. Our platforms are currently focused on data from *A. thaliana*, but we plan to develop a flexible application programming interface (API), such that anyone could build their custom platforms for their individual species.

## Notes

URLs: https://arapheno.1001genomes.org and https://aragwas.1001genomes.org
